# Ultra-Restrictive Opioid Prescription Protocol After Inflatable Penile Prosthesis

**DOI:** 10.7759/cureus.104448

**Published:** 2026-02-28

**Authors:** Joshua M Lohri, Carly Ulrich, Rebecca Spinaris, Jacqueline C Fannin

**Affiliations:** 1 Urology, Charleston Area Medical Center, Charleston, USA; 2 Urology, University of Kentucky, Lexington, USA; 3 Urology, East Carolina University Health, Greenville, USA

**Keywords:** inflatable penile prosthesis, multi-modality pain management, opioids, pain management, penile prosthesis

## Abstract

Background

Addressing the opioid epidemic in the United States requires minimizing the overprescription of opioid pain medications, which poses a challenge for urologic surgeons, as postoperative pain is common after procedures such as inflatable penile prosthesis (IPP) placement. This research aimed to evaluate the impact of an ultra-restrictive opioid prescription protocol (UROPP) on pain management and opioid usage following IPP implant surgery.

Methodology

Patients undergoing IPP surgery were treated perioperatively using a UROPP with 500 mg acetaminophen and 15 mg intravenous ketorolac every six hours and opioids as needed for breakthrough pain. At discharge, some patients were given a three-day prescription of 5 mg oxycodone/325 mg acetaminophen, and all patients were given prescriptions for seven-day supplies of acetaminophen (500 mg) and ibuprofen (600 mg). When IPP surgery was performed as a same-day procedure due to COVID-19 precautions, all patients were given a three-day prescription of 5 mg oxycodone/325 mg acetaminophen at discharge. The patients on the UROPP were compared with a retrospective cohort of IPP patients treated before the institution of the UROPP. Outcomes assessed were opioid use, including total morphine milligram equivalents (MMEs), the number of opioid pills prescribed, and the need for refills or postoperative emergency visits.

Results

In total, 96 patients were analyzed (46 pre-UROPP, 50 post-UROPP) with no significant differences in baseline demographics. UROPP implementation significantly reduced opioid prescribing at discharge (100% vs. 36%, p < 0.001), median opioid pills (19.0 vs. 0.0, p < 0.001), and total MME. There was no increase in 30-day emergency department visits. Inpatient UROPP patients had the greatest reduction, with 96% discharged without opioids and low refill rates, while outpatient UROPP patients had higher refill rates but remained significantly lower than pre-UROPP levels.

Conclusions

The UROPP seems feasible and effective in reducing potentially unnecessary opioid prescribing and the risk of abuse following IPP placement.

## Introduction

Physicians may contribute to the opioid epidemic through routine utilization and potential overprescribing of narcotic pain medications. From 1991 to 2011, the annual number of opioid prescriptions in the United States increased from approximately 76 million to over 200 million [1]. Prescription opioids were implicated in more than 17,000 overdose-related deaths in 2017 alone [2]. During the COVID-19 pandemic, overdose deaths further escalated, with more than 90,000 Americans dying from drug overdoses during peak pandemic years [3]. The pandemic introduced additional challenges to postoperative pain management, including efforts to minimize time spent in healthcare facilities and reduce return visits, potentially influencing opioid prescribing practices [4].

Although postoperative opioid prescribing has contributed to the opioid crisis, postoperative pain can often be effectively managed with little to no reliance on opioids [5]. Changing prescriber habits has therefore become a major focus of national efforts to curb opioid overuse. However, limiting opioid pain medication postoperatively presents unique challenges for urologic surgeons, as postoperative pain is an unavoidable consequence of many urologic procedures. Urologists may inadvertently contribute to opioid overuse through routine prescribing practices following procedures such as inflatable penile prosthesis (IPP) placement [6].

A 2011 study demonstrated that only 58% of prescribed opioid pain medication was consumed following urologic surgery, with 67% of patients retaining excess opioids from their initial prescription [1]. Given the substantial volume of unused opioids, institutions have increasingly adopted strategies to reduce unnecessary prescribing. The field of urology has recognized the importance of appropriate opioid stewardship [6], leading to the implementation of restrictive and ultra-restrictive opioid prescribing protocols (UROPPs) [7-12]. Mark and colleagues demonstrated that an ultra-restrictive opioid protocol following gynecologic and abdominal surgery significantly reduced opioid prescribing at discharge without increasing refill requests or compromising pain control [12].

Based on these findings, we implemented a UROPP to reduce unnecessary narcotic prescribing among patients undergoing IPP placement and evaluated its impact at our institution. Following COVID-19-related precautions that necessitated a transition to same-day IPP surgery, the protocol was modified and its impact reassessed.

## Materials and methods

At a single tertiary care center, a post-implementation cohort was formed with patients who underwent IPP placement after the implementation of UROPP from February 2019 to October 2020. A pre-implementation control consisting of patients who underwent IPP from September 2016 to September 2018, before UROPP adoption, was used for comparison. Exclusion criteria included chronic opioid or non-steroidal anti-inflammatory drug use and stage III kidney disease to ensure a homogenous patient population. A patient list was created from the inclusion and exclusion criteria from the institution’s data warehouse. Electronic medical records were retrospectively reviewed, and data were extracted from the patients’ electronic medical records for analysis. All IPP procedures were performed by the same surgeon with the same perioperative care plan for both cohorts. The study was approved by the Institutional Review Board (approval number: 20-650), and all procedures were in accordance with the ethical standards of the Helsinki Declaration developed by the World Medical Association.

In the pre-implementation cohort, pain management for IPP patients was 5 mg hydrocodone for a Visual Analog pain scale [13] rating of 1-5 and 10 mg hydrocodone for a pain scale rating of 6-10. For breakthrough pain, sudden and intense pain occurring during standard pain management, 2 mg of morphine intravenously was administered every four hours as needed. Postoperative pain management post-implementation of the UROPP for admitted patients involved scheduled acetaminophen (500 mg every six hours) and intravenous ketorolac (15 mg every six hours), with opioids provided only as needed. Patients who required fewer than five opioid doses during hospitalization were discharged without an opioid prescription but received a seven-day supply of 500 mg acetaminophen and 600 mg ibuprofen. Patients requiring more than five opioid doses were discharged with a three-day supply (12 tablets) of 5 mg oxycodone/325 mg acetaminophen, along with non-opioid analgesics.

Due to COVID-19 restrictions, IPP procedures transitioned from inpatient to outpatient settings, leading to an increase in post-discharge opioid prescriptions due to the lack of intravenous pain control in the outpatient setting. The restrictions also limited follow-up duration and postoperative monitoring comparisons between the cohorts. Opioid refill rates and emergency department (ED) visits 30 days postoperatively were only monitored.

Each patient’s acetaminophen dosage was monitored to be below the recommended daily allowance for acetaminophen, which is 4,000 mg per day. The study assessed whether the UROPP remained effective despite these procedural modifications in a subgroup analysis of inpatient and outpatient UROPP patients.

SPSS version 29.0 (IBM Corp., Armonk, NY, USA) was used for all statistical analyses. Continuous variables are reported as means with standard deviations, while categorical variables are reported as numbers with percentages. To determine statistical difference, a two-sided Fisher’s exact test was used for categorical data. Mann-Whitney U tests were used to determine the difference between continuous variables. There were no missing data for variables included in the primary and secondary analyses; no imputation or missing data handling procedures were applied. A significance level of p < 0.05 was considered statistically significant in all comparisons.

Primary outcomes included the proportion of patients discharged with opioid prescriptions, the total number of opioid pills prescribed, and morphine milligram equivalent (MME) at discharge. Secondary outcomes included opioid refill rates and ED visits 30-days postoperatively.

## Results

Cohort characteristics of IPP patients before and after UROPP

A total of 96 patients were analyzed: 46 patients in the pre-implementation UROPP and 50 patients in the post-implementation UROPP cohort. In the pre-implementation cohort without the UROPP, patients were white (89%) with a median age of 68.0 years, and 7% of the patients had a prior IPP. When compared to patients post-implementation of UROPP, there were no significant differences in median age (72.0 and 68.0 years, p = 0.09), race (86% and 95% white, p = 0.53), or prior implant history (4% and 14%, p = 0.38) in the inpatient and outpatient UROPP cohorts, respectively, as well as the pre-implementation cohort without UROPP.

Patient postoperative opioid use before and after UROPP

Following UROPP implementation, the percentage of patients discharged with opioids significantly decreased from 100% to 36% (p < 0.001). Additionally, the median number of opioid pills prescribed decreased from 19.0 (interquartile range (IQR) = 8.0) to 0.0 (IQR = 12.0) pills (p < 0.001), and total MME at discharge decreased from 0.0 MME (IQR = 60.0) to 7.5 MME (IQR = 3.8) (p = 0.02) with implementation of UROPP. There was also no significant increase in 30-day postoperative ED visits following UROPP implementation in admitted IPP patients (4% vs. 11%, p = 0.65).

Comparison of patients discharged with opioids before and after UROPP implementation

A subgroup analysis comparing inpatient (n = 28) and outpatient UROPP (n = 22) to pre-UROPP patients found a significant decrease in opioid use for inpatient IPP by the reduction of prescribed opioids at discharge, number of opioid pills, and total MME (Table 1). Only 4% of admitted UROPP patients requested refills, with 96% of patients being discharged without opioid prescriptions. The shift to outpatient IPP placement due to COVID-19 increased the number of opioid prescriptions at discharge (23%); however, opioid use remained significantly lower than pre-UROPP levels (100%; p < 0.001). Notably, outpatient UROPP patients had an increased percentage for refills (23% vs. 4%) compared to inpatient UROPP patients (p < 0.0001) (Table 1).

**Table 1:** Pain management in IPP patients with UROPP before and after COVID-19. *: The outpatient cohort represents the change from inpatient to outpatient IPP due to the COVID-19 pandemic. ^†^: Fisher’s exact test was used to determine statistical significance; ^#^: Mann-Whitney U test was used to determine statistical significance n: number; IPP: inflatable penile prosthesis; UROPP: ultra-restrictive opioid pain protocol; PACU: post-anesthesia care unit; IQR: interquartile range; NA: no test statistic available

	UROPP		P-value	Test statistic
Opioid prescribing characteristics	Inpatient	Outpatient		
	(n = 28)	(n = 22)		
Opioid prescribed at discharge^†^, n (%)				NA
Yes	1 (4)	17 (77)	<0.0001	
No	27 (96)	5 (23)		
Number of opioid doses after PACU^#^, median (IQR)	1.0 (4.0)	1.0 (6)	0.73	291
Number of opioid pills^#^, median (IQR)	0.0 (0.0)	12.0 (8.0)	<0.001	535
Total MME^#^, median (IQR)	0.0 (0.0)	60.0 (58.8)	<0.001	527.5
Prescription filled^†^, n (%)				NA
Yes	1 (4)	15 (88)	<0.0001	
No	27 (96)	2 (12)		

To assess the difference in opioid use in discharged patients with opioid prescriptions after transitioning to outpatient IPP procedures due to COVID-19, a subgroup analysis was performed with IPP patients discharged before and after UROPP adoption (Table 2). There was a significant reduction in the median number of opioid pills (19.0 pills, IQR = 8.0 vs. 12.0 pills, IQR = 0.0, p < 0.001) and median total MME (150.0 MME, IQR = 83.0 vs. 68.0 IQR = 20.0, p < 0.001), respectively.

**Table 2:** Pain management in patients discharged with opioids before and after implementation of UROPP. ^†^: Fisher’s exact test was used to determine statistical significance; ^#^: Mann-Whitney U test was used to determine statistical significance. *: The UROPP cohort represents both inpatient and outpatient UROPP patients. UROPP: ultra-restrictive opioid pain protocol; n: number; IQR: interquartile range; MME: morphine milligram equivalents; NA: test statistic not available

Opioid prescribing characteristics	Before		P-value	Test statistic
	UROPP	UROPP		
	(n = 46)	(n = 17)		
Opioid prescribed at discharge^†^, n (%)				NA
Yes	46 (100)	17 (100)	1	
No	0 (0)	0 (0)		
Number of opioid pills^#^, median (IQR)	19.0 (8.0)	12.0 (0.0)	<0.001	88.0
Total MME^#^, median (IQR)	150.0 (83.0)	68.0 (20.0)	<0.001	0

## Discussion

The findings of this study demonstrate that implementation of a UROPP significantly reduced opioid prescriptions and overall opioid exposure in patients undergoing IPP placement without compromising postoperative pain control. Importantly, reductions in opioid prescribing were not associated with increased emergency department visits, suggesting that adequate pain management was maintained. Although the transition to outpatient IPP surgery during the COVID-19 pandemic resulted in a modest increase in opioid prescriptions at discharge, prescribing levels remained significantly lower than those observed before UROPP implementation.

This protocol is particularly relevant in West Virginia, a region consistently described as the epicenter of the opioid epidemic [14]. For more than a decade, West Virginia has reported the highest overdose death rates in the United States, compounded by substantial social and health disparities and generational substance misuse [15]. Patients in Appalachia undergoing surgical procedures face heightened risks of persistent or chronic postoperative opioid use due to a convergence of risk factors, including tobacco use, antidepressant use, preoperative pain, lower socioeconomic status, male sex, older age, multiple medical comorbidities, and histories of mental health disorders or substance use [14,16-18]. In such high-risk populations, establishing a practical standard of care for opioid stewardship is essential.

Urologic procedures have been linked to persistent opioid use, with patients receiving opioids after artificial urinary sphincter placement demonstrating a threefold increase in chronic opioid use [19]. While some risk factors may be mitigated through patient education, surgeons play a critical role in reducing opioid exposure through protocol-driven prescribing. Multiple studies have shown that reduced or opioid-free postoperative regimens are feasible for urologic procedures such as ureteroscopy and retrograde intrarenal surgery [20]. However, standardized opioid-restrictive protocols specific to IPP placement have not been widely established [21].

Previous studies utilizing multimodal analgesia protocols for IPP implantation demonstrated reductions in opioid requirements but often relied on extended non-opioid regimens, gabapentinoids, nerve blocks, or prolonged discharge prescriptions [22-24]. The UROPP implemented in this study achieved comparable reductions in opioid use while limiting discharge prescriptions to significantly shorter durations and avoiding additional controlled substances or invasive analgesic techniques. This approach may reduce patient burden, financial costs, and exposure to medications with misuse potential, including gabapentinoids.

Additionally, the use of intravenous ketorolac within the UROPP aligns with evidence demonstrating reduced narcotic requirements following urologic surgery without compromising safety [18]. Although pain scores were not assessed using a Visual Analog Scale, this study prioritized clinically meaningful outcomes such as unplanned visits, refill requests, and total opioid exposure. Prior studies have shown worse postoperative pain among diabetic IPP patients [22], though the high prevalence of diabetes in this cohort suggests the protocol remains effective across common comorbidities.

The shift to same-day discharge during the COVID-19 pandemic introduced additional considerations. While outpatient IPP procedures required modestly higher discharge prescriptions, opioid exposure remained substantially lower than historical levels. Similar transitions to same-day discharge have been reported for other urologic procedures, including robotic prostatectomy and percutaneous nephrolithotomy, though some studies have noted increased emergency department visits related to postoperative pain [25,26]. Notably, such increases were not observed in the present study.

Limitations include the single-institution design, small subgroup sample sizes, retrospective comparison cohorts, and lack of detailed quality-of-life metrics. Given that this study was performed in a high-risk opioid region, the generalizability of the findings warrants consideration. However, because the intervention modifies standardized prescribing practices rather than patient-level characteristics, its applicability may extend beyond regions with elevated opioid misuse. The absence of increased unplanned care or refill requests in this high-risk cohort supports the safety of broader implementation. Future multicenter studies with larger cohorts and long-term follow-up in varied geographic and risk settings are warranted to validate these findings and further evaluate patient-reported outcomes. The retrospective pre-post design introduces potential temporal bias, as evolving institutional practices, increased national awareness of opioid stewardship, and COVID-19-related systemic changes may have influenced prescribing independent of protocol implementation. While these secular trends cannot be fully accounted for, the sustained reductions in opioid prescribing without increased unplanned care suggest a meaningful association with the ultra-restrictive protocol.

## Conclusions

UROPP implementation significantly reduced opioid prescriptions and overall opioid exposure following IPP placement without negatively impacting pain management or increasing emergency department utilization. Even with the transition to outpatient surgery, opioid prescribing remained well below pre-UROPP levels. Broader adoption of similar opioid-restrictive protocols may play a critical role in addressing postoperative opioid exposure and mitigating long-term opioid dependence in urologic surgery.
